# A 28 nm Bulk-CMOS Analog Front-End for High-Rate ATLAS Muon Drift-Tube Detectors

**DOI:** 10.3390/s20010042

**Published:** 2019-12-19

**Authors:** Alessandra Pipino, Federica Resta, Luca Mangiagalli, Marcello De Matteis, Hubert Kroha, Robert Richter, Oliver Kortner, Andrea Baschirotto

**Affiliations:** 1 Depterment of Physics, University of Milano-Bicocca, Piazza Della Scienza 3, 20123 Milan, Italy; alessandra.pipino@unimib.it (A.P.); federica.resta@unimib.it (F.R.); marcello.dematteis@unimib.it (M.D.M.); andrea.baschirotto@unimib.it (A.B.); 2 Max-Planck-Institute for Physics, Foehringer Ring 6, D-80805 Muenchen, Germany; kroha@mpp.mpg.de (H.K.); richterr@mppmu.mpg.de (R.R.); kortner@mpp.mpg.de (O.K.)

**Keywords:** ATLAS, reset, dead-time, read-out electronics, front-end, 28 nm CMOS technology

## Abstract

This paper presents the design and experimental characterization of a 28 nm Complementary Metal Oxide Semiconductor (CMOS) Analog Front-End (AFE) for fast-tracking small-diameter Muon Drift-Tube (sMDT) detectors. The device exploits an innovative analog signal processing that allows a strong increase in the detection rate of events and significantly reduces the impact of fake/pile-up events, which often corrupt incident radiation energy events. The proposed device converts the input charge coming from incident radiations into voltage by a dedicated Charge-Sensitive Preamplifier (CSPreamp). Therefore, the fast-tracking concept relies on sampling the slope of the CSPreamp output voltage and using it for detecting both the incident event arrival instant and the amount of charge that has been effectively read out by MDT detectors. This avoids the long processing times intrinsically needed for baseline recovery transient, during which the detected signal can be severely corrupted by additional and unwanted extra-events, resulting in extra-charge (and thus in CSP output voltage extra-transient) during the signal roll-off. The proposed analog channel operates with a 5–100 fC input charge range and has a maximum dead-time of 200 ns (against the 545 ns of the state-of-the-art). It occupies 0.03 mm^2^ and consumes 1.9 mW from 1 V of supply voltage.

## 1. Introduction

Periodic upgrades of the Large Hadron Collider (LHC) to discover novel physics constantly generate new technological challenges. The upgrade of the read-out electronics for particle detectors is a fundamental technological step for Phase-II of High Luminosity-LHC (HL-LHC). Luminosity is expected to increase by an order of magnitude with respect to Phase-I, with a consequent interaction rate increase. This pushes towards the development of faster read-out integrated circuits able to perform fast analog/digital signal processing. For these reasons, research in high-energy physics integrated circuits is pushing towards ultra-scaled technologies (such as 28 nm) with significant benefits in terms of both speed and radiation hardness [1,2].

Muon Drift-Tube (MDT) chambers (currently installed in ATLAS (A Toroidal LHC ApparatuS) [3]) employ a dedicated read-out analog front-end (called ASD, Amplifier-Shaper-Discriminator) based on the cascade of a Charge-Sensitive Preamplifier (CSPreamp), a continuous-time Shaper and a Discriminator stage [4].

When an incident radiation drives a certain amount of charge, the CSPreamp quickly rises up, converting the charge into voltage. Afterwards, the Shaper adapts the CSPreamp output voltage into a bipolar pulse [4,5] signal that allows a robust and slow baseline recovery, typically lasting about 500 ns.

**Figure 1:**
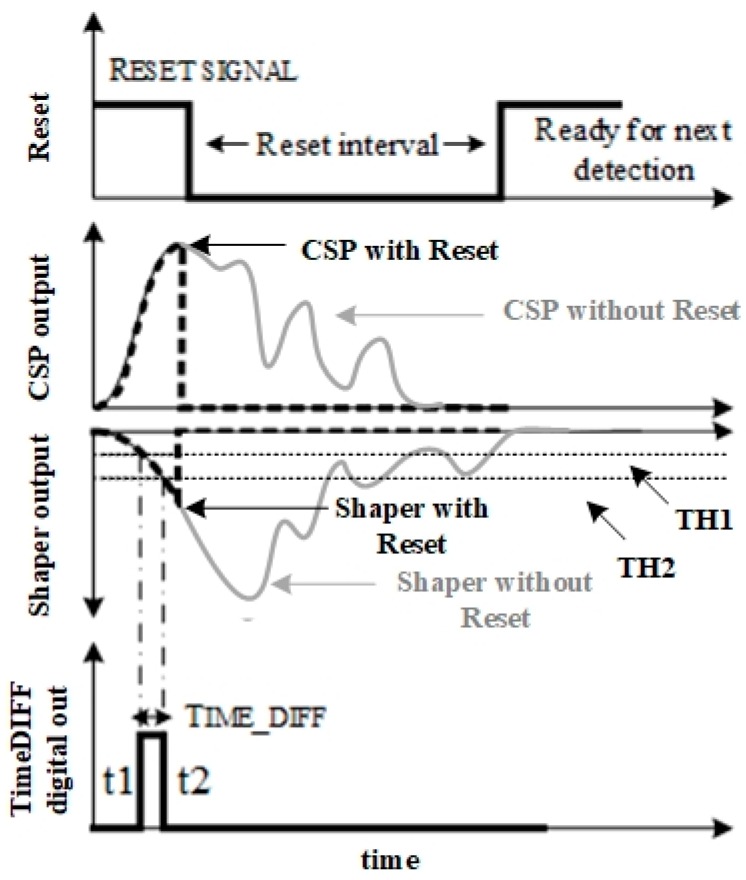
Fast-tracking operation principle.

If, during the baseline recovery time, other spurious events hit the detector, then the CSPreamp output voltage modifies the output voltage time realization following a transient, which is also a function of these last recently arrived events, as it is also shown in Figure 1.

For these reasons, the classical read-out analog front-end for MDT detectors adopts a dead-time status, during which the system is waiting for the signal processing to end and it is not able to detect novel events [4].

After amplification and shaping, conversion into the digital domain is performed with a dedicated Time-to-Digital Converter (TDC) [5,6,7,8]. The ASD returns two important time-domain data for each event: the charge arrival time and the amount of charge by Time-over-Threshold (ToT) encoding.

This is a robust and reliable solution for small-diameter MDT (sMDT) detectors, whereas it scarcely complies with Phase-II upgrades speed requirements, for several reasons, among them:

- Both devices presented in references [4,6] have been developed in very old CMOS processes, so they have lower transition frequency compared to nm-range technologies.
- The intrinsic necessity for a dead-time scarcely fits with the fast response requirements imposed by HL-LHC.

The main causes that generate signal pile-up events in MDT detectors are secondary spurious pulses (SSPs) [8] generated by stochastic ionization clusters in the gas. In order to limit the amount of threshold crossing generated by SSPs, the read-out electronics adopt different strategies: CSPreamp small time constants (large bandwidth), bipolar shaping and the dead-time status after every event detection to neglect the following peaks 4.

This avoids a pile-up of spurious signals on the first pulse, which is usually the interesting one. The dead-time should last long enough to avoid the detection of non-interesting electrons drifting in the tube after the first ionization. The maximum drifting time depends on many factors and is about 500 ns for MDT.

Small-diameter MDT (sMDT) chambers [9] will replace the previous ones in ATLAS at HL-LHC to meet the rate capability requirement of Phase-II. These upgraded chambers are built with tubes of smaller diameter, allowing higher rate capability due to the reduction of drift time to about 180 ns. In this situation, the muon efficiency is not limited by detector drift time, but it is limited by pile-up effects [10]. Indeed, superposition of signals from different events decreases the efficiency and accuracy of threshold crossing time and the spatial resolution of the drift tubes. 

Reference [11] reports a preliminary description of this technique based on rough simulation results of the fast-tracking concept. Reference [12] addresses the design and integration of a dedicated logic circuit core responsible for reset and pulse pile-up rejection in multi-channel spectroscopic-grade Application Specified Integrated Circuits (ASICs). This work gives several details about the logic circuit design and characterization, whereas it lacks additional and relevant information about the impact of the reset action on the operation Preamplifier and Shaper stages (basically, the analog front-end transistor-level design is not reported). 

Reference [13] presents complete readout and trigger electronics, based on the waveform digitization and pipeline readout, for the KOTO experiment at J-PARC, Japan. The front-end is based on commercial off-the-shelf components and adopts a 10^th^-order Bessel filter to smooth the pile-up peaks. Effectively, this solution appears very robust, even if application-specified integration in silicon is critical due to the large power per unit area to be allocated to very high-order analog filters. This justifies the off-the-shelf approach for read-out electronics implementation.

The hereby proposed front-end totally changes the state-of-the-art paradigm in MDT read-out Analog Front-End (AFE) by proposing an innovative technique, integrated in nm-range technologies for the first time, based on sampling the slope of the CSPreamp output voltage and then resetting/forcing to common-mode voltage both the CSPreamp and Shaper [14] output voltages after the slope sampling.

This avoids waiting for a complete baseline recovery and significantly enhances the speed of the system.

Thus, the device presented in this paper advances the state-of-the-art because:

- it scales down the integration of ATLAS sMDT analog signal processing stages from 0.13 µm@3.3 V to 28 nm@1 V [6];
- it increases the event count rate thanks to a shorter deadtime (<200 ns) after the first relevant pulse;
- it reduces power consumption with respect to the state-of-the-art pile-up rejection techniques;
- it embeds an external priority reset feature that increases the controllability of the system from an external environment.

This paper is organized as follows: the most relevant system and transistor-level design aspect are presented in Section 2, Section 3 shows time-domain experimental measurements from the silicon prototype, and at the end of the paper, conclusions are drawn.

## 2. Fast Read-Out Architecture

**Figure 2:**
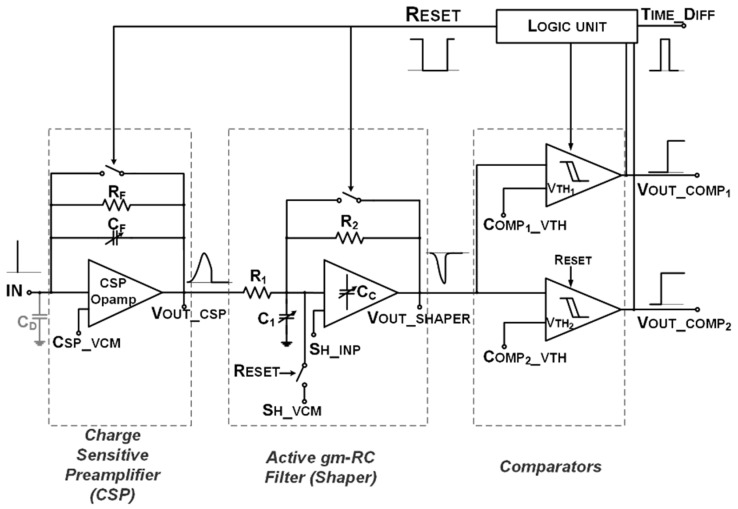
Fast-Tracking Front-End (FTfe) system-level/circuital top-view.

Figure 1 and Figure 2 show the simplified block diagram and the principle of operation of the proposed read-out channel.

The presented Fast-Tracking Front-End (FTfe) device compares the CSPreamp output voltage with two reference threshold voltages and extracts both arrival time and charge information from signal slope. As soon as the reset signal is disabled, the front-end restarts to detect and process the next incoming signal. Thanks to the reset operation, the long tail of the sMDT signal does not affect the charge Preamplifier and Shaper operations.

The FTfe device detects the input charged particles coming from sMDT detectors and efficiently extracts information from the signal slope in terms of arrival time and amount of charge. The analog part of the channel includes a CSPreamp and the analog Shaper based on Active-G_m_-RC low-pass biquadratic cell (both devices have a proper reset operation).

The CSPreamp output voltage is amplified and shaped before the comparison with two different off-chip threshold voltage references (V_TH1_ and V_TH2_). Detection of the threshold crossing time instants (by COMP_1_ and COMP_2_) allows the sampling of the signal slope and the encoding of such a slope by the time-width of the resulting digital pulse.

V_TH1_ crossing time corresponds to the charge arrival time. Notice that if V_TH2_ is never crossed, COMP_1_ provides Time-over-Threshold (ToT) information.

In nominal conditions, both thresholds are crossed, both comparators provide specific output pulses (V_OUT_COMP1_ and V_OUT_COMP2_), and thus the Logic Unit receives such an input digital pulse and provides two digital output signals: RESET and TIME_DIFF.

The RESET signal forces both the CSPreamp and Shaper output voltages towards common-mode dc voltage. This has the following main advantages:

- It avoids multiple threshold crossings due to eventual pile-up distortion.
- It allows the detection of the first pulse coming from charge induced by the first incident ionization, ignoring the spurious pulses (shown in grey trace in Figure 1).
- It increases the maximum count rate, which is no longer forced to wait for baseline restoration time.

The TIME_DIFF pulse time-width (shown in Figure 2) is the difference between two crossing instants. This allows the changing of the range of TIME_DIFF values for a given input range by just changing V_TH1_ and V_TH2_, as shown in the following equation:

(1) $${\mathrm{TIME}}_{\mathrm{DIFF}}={\;t}_{2}-t_{1}=\frac{V_{\mathrm{TH}2}-V_{\mathrm{TH}1}}{\mathrm{SR}}$$

where t_1_ and t_2_ are the threshold crossing instants, V_TH1_ and V_TH2_ are the threshold voltages defined with respect to the signal baseline, and SR is the Shaper output voltage slew-rate, which depends on input charge Q_IN_.

An interesting aspect of this readout technique is that V_TH1_ is set in order to be clearly separated from noise power (>3·σ_NOISE_, where σ_NOISE_ is the noise power), while V_TH2_ is set in order to meet the TIME_DIFF duration specifications. In particular, for the sMDT data acquisition system, the TIME_DIFF width must be higher than 1 ns for maximum input charge (Q_IN_) in order to fit with the clock frequency of the following off-chip Time-to-Digital Converter [4].

This system allows a strong reduction of event detection time, decreasing the probability of pile-up. The design has been done considering 10 pF of detector capacitance and 5–100 fC of input charge range [11]. Each block is described in detail in the next subsections.

### 2.1. Charge-Sensitive Preamplifier

**Figure 3:**
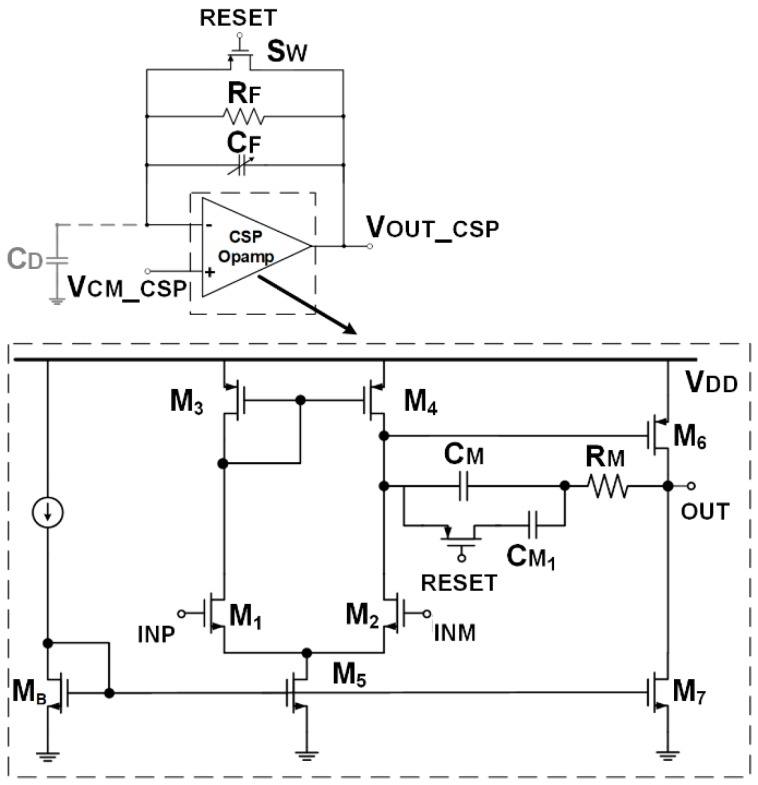
Charge-Sensitive Preamplifier (CSPreamp) and Operational Amplifier (opamp) transistor-level scheme.

The CSPreamp circuital topology is shown in Figure 3, based on classical feedback charge sensing [15]. The feedback resistor R_F_ cancels the injected charge from C_F_ and allows the integration of the following event.

The higher R_F_ is, the more accurate is the integration phase, but the longer the time for baseline restoration is, and then the time between two readable events. The lower R_F_ is, the lower the signal read by the CSPreamp is due to the inaccurate charge integration, but the tail is faster, allowing higher input event data-rate. This trade-off is optimized through the reset switch, which reduces the reset time. As indicated in Table 1, R_F_ has been set to 30 kΩ, ensuring high data-rate with very accurate charge integration.

The opamp electrical performances and the detector parasitic capacitance C_D_ affect the CSPreamp output voltage in terms of gain (i.e., peak voltage) and time constants (i.e., peaking time and rise/fall time) [6]. The most important parameter values for both CSPreamp and Shaper are listed in Table 1.

The opamp, shown in Figure 3 is a two-stage Miller-compensated amplifier. The 28 nm bulk-CMOS process forces input MOS transistors to operate in sub-threshold region, assuming 1 V supply voltage and 0.55 V transistors’ threshold voltage. This situation can be exploited to maximize the input g_m_ (i.e., the sensitivity) and satisfy the noise requirement.

The input g_m_ is 5 mA/V with a bias current of 480 µA; the resulting input transistors have a W/L of 180 µm/300 nm. The Miller capacitance C_M_ is equal to 1 pF, while the nulling Right-Half-Plane Zero resistor R_M_ value is 250 Ω. In order to improve the phase margin during the reset transitions (in which the CSPreamp operates as a unity gain buffer with maximum loop-gain), a 5 pF programmable capacitance controlled by the same reset signal has been added in parallel with C_M_, increasing the Miller effect.

A programmable C_F_ has been implemented in order to control the CSP output pulse amplitude (i.e., sensitivity) vs. the larger Process-Voltage-Temperature (PVT) variation of 28 nm technology. A 4-bit capacitor array has been implemented, with 5% accuracy and a nominal capacitance value of 750 fF.

### 2.2. Shaper

**Figure 4:**
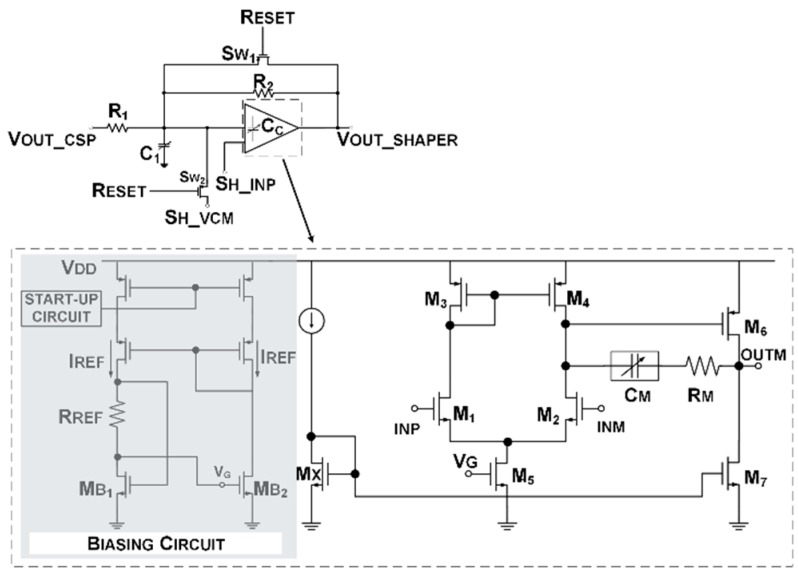
Shaper and opamp transistor-level scheme.

The shaping stage has been realized with an Active-G_m_-RC filter topology [14], as illustrated in Figure 4. It is a biquadratic cell, characterized by a closed-loop structure that exploits the opamp as open-loop integrator. Two switches (S_W1_ and S_W2_) have been embedded for reset purposes to reduce charge injection. The transfer function of the Active-G_m_-RC can be expressed as follows:

(2) $$T\left(s\right)=\frac{G}{\left(\frac{s^{2}}{\omega_{0}^{2}}+\frac{s}{\omega_{0}Q}+1\right)}$$

where

(3) $$G=\frac{R_{2}}{R_{1}},\;\omega_{0}=\sqrt{\frac{\omega_{op}}{C_{1}R_{2}}},\;Q=\frac{\sqrt{\omega_{op}C_{1}R_{2}}}{\left(1+G\right)}$$

Opamp unity-gain-frequency (UGB) is comparable with the desired filter pole frequency. This solution strongly reduces its power consumption with respect to standard closed-loop structures, in which the opamp UGB should be much higher than the filter pole. To comply with the specifications reported in [11], a pole frequency at about 7 MHz and gain at 20 dB have been chosen. The biquadratic cell design parameters are listed in Table 1.

The opamp is based on the class-A Miller scheme, as shown in Figure 4. The input stage MOS differential pair works again, as in CSPReamp, in the sub-threshold region, because of the reduced [V_DD_-V_TH_] space of the 28 nm technology.

A dedicated bias circuit (grey box of Figure 4) matches the input stage g_m_ with an external resistor [14], in order to track the integrated resistor process deviation and to improve the accuracy of cut-off frequency calibration.

Through the matching between M_B1_ (W/L = 9 µm/300 nm) and the opamp input transistors M_1_ and M_2_ (W/L = 30 µm/300 nm), the input g_m_ is forced to be proportional to 1/R_REF_, where R_REF_ is matched with the integrated resistances of the feedback net. In this design, R_REF_ is about 800 Ω, M_B1_ aspect ratio is three times lower than M_5_ aspect ratio (W/L = 30 µm/300 nm) and the bias current is equal to 100 µA.

This g_m_-tracking approach allows full spread calibration of the filter pole by acting only on passive components. Moreover, all the capacitors of the shaping stage (i.e., C_1_ and the opamp Miller capacitance C_M_) have been replaced with 5-bit programmable capacitor arrays. In this way, the overall filter frequency response can be controlled to compensate for the cut-off frequency (peaking time and tail cancellation) variations due to the large 28 nm technology PVT variations.

### 2.3. Comparators and Logic Block

The two comparators are an un-compensated version of the opamp used in CSPreamp. Their dynamic power consumption is 100 µA and they are turned off, disconnecting the supply during the reset phase.

**Figure 5:**
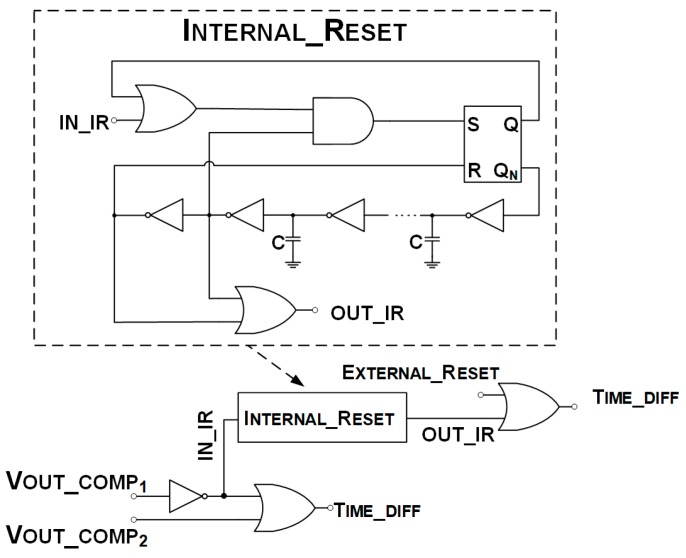
Digital circuit for TIME_DIFF and RESET pulses generation.

The voltage thresholds are externally controlled by a voltage generator, and the RESET signal is generated from V_OUT_COMP1_ using the logic circuit as shown in Figure 5. The reset is fixed in the absence of an external reset.

The TIME_DIFF signal is provided by a NOR logic port, which receives the V_OUT_COMP1_ inverted digital value and V_OUT_COMP2_. It is also possible to trigger the channel reset using an external signal, as illustrated in Figure 5.

The external reset allows the changing of the reset duration, a key aspect for this application, where the experiment may require externally increasing the dead-time after the first peak.

## 3. Experimental Results

The proposed FTfe device has been integrated in 28 nm bulk-CMOS technology, which has been selected for better rad-hard robustness [2].

Channel area is 0.03 mm^2^ and power consumption is 1.9 mW from a 1 V supply, for one channel.

**Figure 6:**
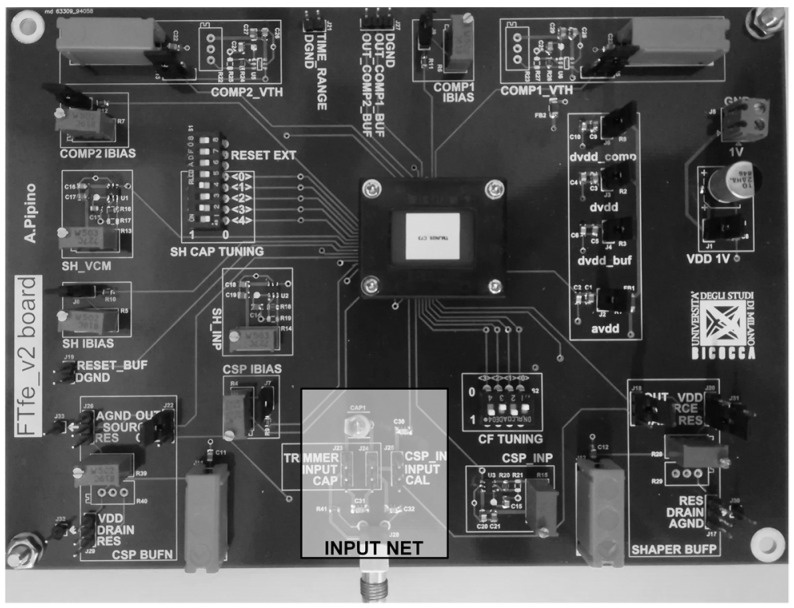
Printed Circuit Board (PCB) for FTfe lab testing.

A custom Printed Circuit Board (PCB), whose photo is shown in Figure 6 has been designed and implemented for testing the CMOS FTfe silicon prototype.

The input net (grey box in Figure 6) implemented on the PCB emulates the sMDT detector providing a programmable current pulse amplitude at the CSPreamp input, enabling the possibility to test the time-domain response of the FTfe sweeping the input charge.

The measurements have been done in the worst-case scenario for the TIME_DIFF, i.e., for maximum Q_IN_ that is 100 fC, and it is implemented with a current pulse of 40 µA amplitude and 2.5 ns time-width.

**Figure 7:**
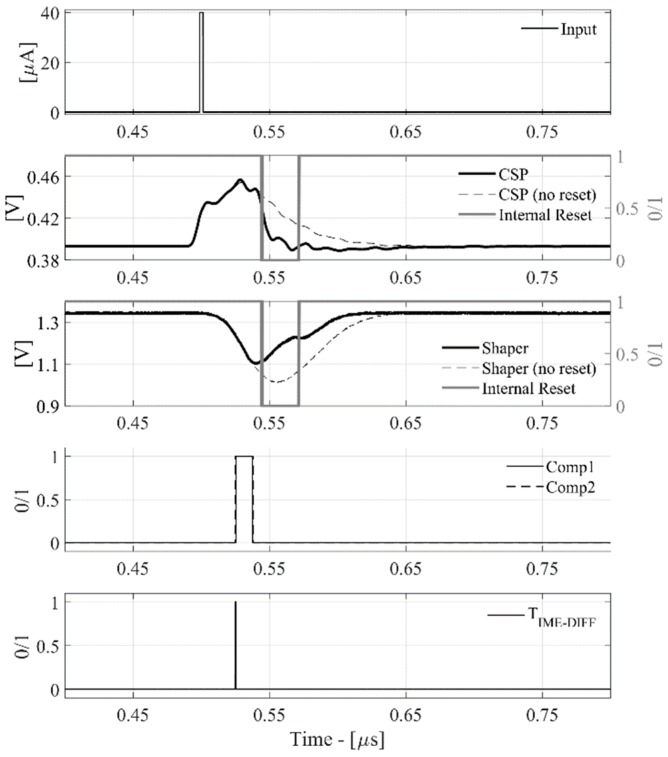
Input pulse, CSPreamp, Shaper, internal reset, comparators, TIME_DIFF signals for Q_IN_ = 100 fC.

**Figure 8:**
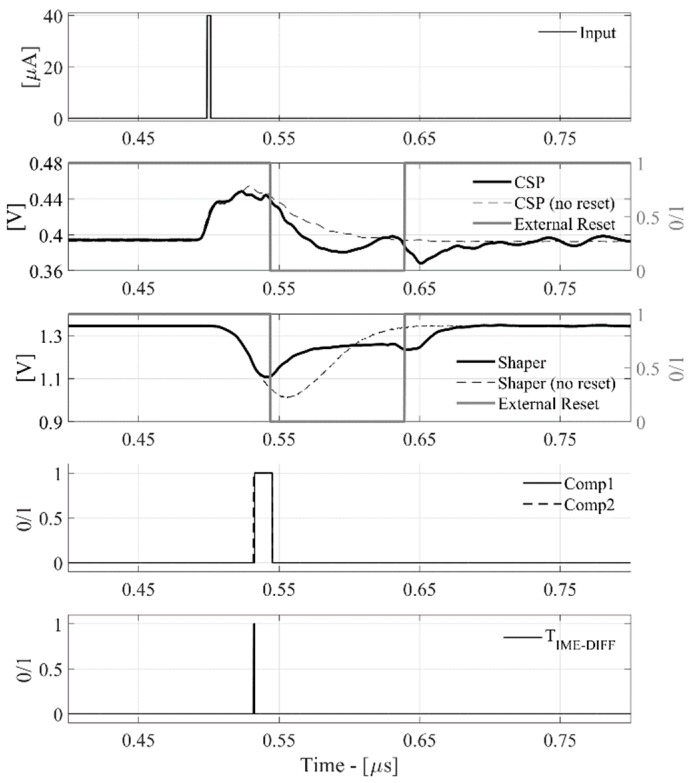
Input pulse, CSPreamp, Shaper, external reset, comparators, TIME_DIFF signals for Q_IN_ = 100 fC.

The relative time-domain waveforms are shown in Figure 7 and Figure 8, highlighting the nominal read-out behavior and its capability to reset, providing an internal or an external reset signal.

In particular, in Figure 7 it is possible to see both CSPreamp and Shaper output signals (feeding a proper analog buffer for probe driving) in two specific conditions: with and without the RESET (dashed lines and solid lines, respectively).

These plots show a reduction in dead-time of about 50 ns with reset enabled. In particular, the internal reset is fixed, and it depends on the logic unit operation.

**Figure 9:**
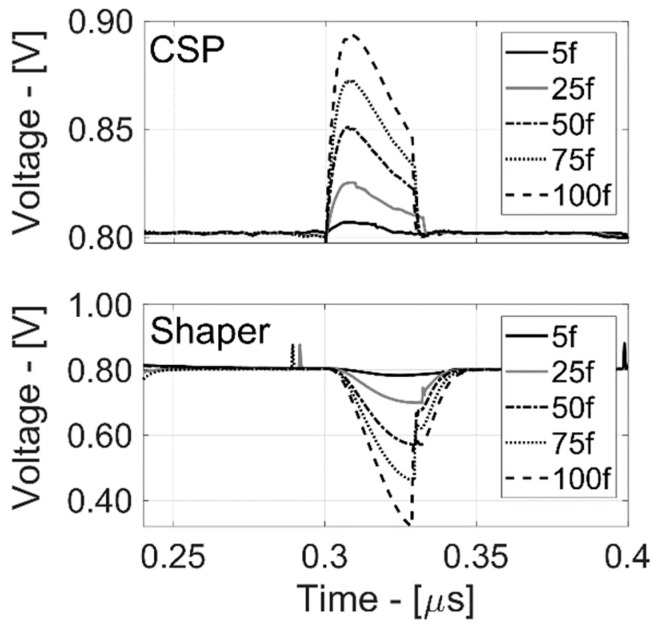
CSPreamp and Shaper output voltages post-layout simulation results vs. input charges.

**Figure 10:**
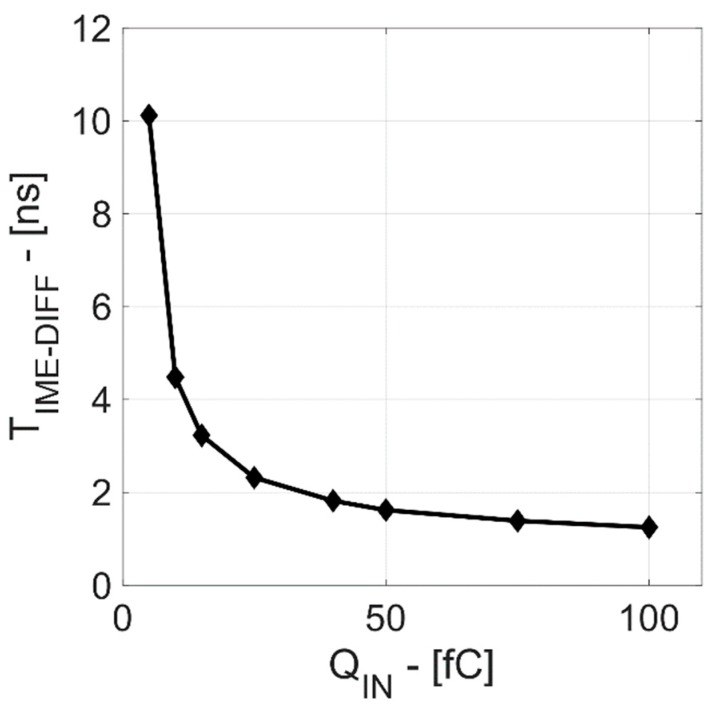
TIME_DIFF vs. QIN (input charge).

In Figure 8, the reset time-width is externally forced by a synchronized voltage pulse. In both cases, the device is able to process the charge, providing the same TIME_DIFF. The measurement taken at Q_IN_ of 100 fC is in good agreement with the post-layout simulations (reported in Figure 9 and Figure 10) in terms of timing signal evolution and TIME_DIFF value.

In order to compare signals obtained from measurements with the characterization obtained from simulations, it is important to consider the effect of the output buffers. Buffer stages used to drive analog output pads were not reset as the internal circuit, and for this reason, their response to the internal reset is limited by their bandwidth. A different baseline value is also given by the buffers.

Comparison with State-of-the-Art

Table 2 compares the proposed FTfe device with current implementations of analog electronics for ATLAS sMDT detectors [4,6] and with other pile-up rejection approaches, which have been adopted in the literature by some important electronics systems in both Application-Specified-Integrated-Circuits [12] and off-the-shelf solutions [13].

Considering state-of-the-art electronics for ATLAS sMDT, the proposed device’s performance shows lower power consumption (sustained by the higher g_m_ in 28 nm technology) and lower area occupancy. This is also due to the detector’s lower parasitic capacitance in FTfe (10 pF against 60 pF in [4,6]), mainly due to the more compact under-development MDT detectors for Phase-II [3]), slightly relaxing the CSPreamp power requirements. Importantly, FTfe significantly reduces the required dead-zone time-width by a factor ×2.65 wrt (with respect to) classical analog front-end [4,6] electronics for sMDT detectors. Moreover, the dead-time reduction is also evident with respect to both dedicated CMOS [12] and off-the-shelf [13] pile-up rejection techniques.

## 4. Conclusions

A Fast-Tracking Front-End integrated in CMOS 28 nm technology node has been hereby presented. The device scales down (from a 0.13 µm to a 28 nm CMOS process) the technology node for analog front-end read-out electronics operating in ATLAS Muon Drift-Tube Detectors. The device strongly mitigates the pile-up distortion effects on the voltage signal, encoding incident charge at the output of the analog front-end. The device also reduces power consumption and area with respect to both classical and advanced (pile-up rejection) techniques.

## Figures and Tables

**Table 1:** CSPreamp and Shaper design parameters.

CSPreamp		Shaper	
Feedback Capacitance—C_F_	750 fF	Input Resistance - R_1_	2 kΩ
Feedback Resistance—R_F_	30 kΩ	Feedback Resistance - R_2_	20 kΩ
Operational Amplifier Output stage Typology	Class A	Ground Capacitance - C_1_	2 pF
Operational Amplifier Input stage Transconductance—g_m_	5 mA/V	Operational Amplifier Output Stage Typology	Class A
Operational Amplifier—DC Gain	42.5 dB	Operational Amplifier Input stage Transconductance - g_m_	1 mA/V
Operational Amplifier Unity Gain Bandwidth—UGB	557 MHz	Operational Amplifier DC Gain	53 dB
		Operational Amplifier Unity Gain Bandwidth - UGB	79 MHz
		Low-Pass -3 dB Frequency - f_p_	168 kHz

**Table 2:** State-of-the-art comparison.

Parameter	This Work	[4]	[6]	[12]	[13]
CMOS Technology	CMOS 28 nm	CMOS 5 µm	CMOS 0.13 µm (High-VT Devices)	CMOS 0.35 µm	Off-the-Shelf
Supply Voltage	1 V	3.3 V	3.3 V	3.3 V	5 V
Area (1 channel Read-Out)	0.03 mm^2^	1.2 mm^2^	0.7 mm^2^	0.0048 mm^2^ (only the Logic Circuits generating the Reset Signal)	−
Power consumption (1 channel Read-Out)	2.6 mW	39 mW	33 mW	−	−
Detector Capacitance	10 pF	60 pF	60 pF	−	−
Input Charge	5–100 fC	5–100 fC	5–100fC	−	−
Maximum Dead Time	200 ns	530 ns	535 ns	40 µs	4 µs
